# Commencement in the Medical Institute

**Published:** 1845-04

**Authors:** 


### COMMENCEMENT IN THE MEDICAL INSTITUTE.

The Catalogue of Graduates in the Medical Institute of Louisville was appended to our March number, from which it was seen that the degree of M.D., in ordinary, was conferred upon seventy-one gentlemen, the honorary degree upon three, and the degree *ad eundem* upon three. This indicates a highly prosperous condition in the school, and yet the number of graduates is hardly as great as might have been expected from a class of two hundred and eighty-six. It will be found that the number of candidates is always smaller, in proportion, in a growing than in a declining school, for the reason that in the former a larger proportion of the class consists of first-course students. Of the gentlemen upon whom the honorary degree was conferred we have the pleasure of a personal acquaintance with two —Dr. Debow, of Hartsville, Tennessee, and Dr. Dawson, of Jamestown, Ohio. The former has distinguished himself by his operations in Surgery, and is well known among his neighbors for his high literary and scientific attainments. The latter has made himself extensively and favorably known by his contributions to the various medical journals of the country.

